# Supplementary material for: Post-Concussion Symptoms in Complicated vs. Uncomplicated Mild Traumatic Brain Injury Patients at Three and Six Months Post-Injury: Results from the CENTER-TBI Study
Source: J Clin Med. 2019 Nov 8;8(11):1921. doi: 10.3390/jcm8111921 (PMC6912209; doi:10.3390/jcm8111921)
Supplement: Supplementary file 1 [file jcm-08-01921-s001.pdf]

File 1

The CENTER-TBI participants and investigators:

Cecilia Åkerlund[1], Krisztina Amrein[2], Nada Andelic[3], Lasse Andreassen[4], Audny Anke[5], Anna Antoni[6], Gérard Audibert[7], Philippe Azouvi[8], Maria Luisa Azzolini[9], Ronald Bartels[10], Pál Barzó[11], Romuald Beauvais[12], Ronny Beer[13], Bo-Michael Bellander[14], Antonio Belli[15], Habib Benali[16], Maurizio Berardino[17], Luigi Beretta[9], Morten Blaabjerg[18], Peter Bragge[19], Alexandra Brazinova[20], Vibeke Brinck[21], Joanne Brooker[22], Camilla Brorsson[23], Andras Buki[24], Monika Bullinger[25], Manuel Cabeleira[26], Alessio Caccioppola[27], Emiliana Calappi [27], Maria Rosa Calvi[9], Peter Cameron[28], Guillermo Carbayo Lozano[29], Marco Carbonara[27], Giorgio Chevallard[30], Arturo Chieregato[30], Giuseppe Citerio[31, 32], Maryse Cnossen[33], Mark Coburn[34], Jonathan Coles[35], Jamie D. Cooper[36], Marta Correia[37], Amra Čović[38], Nicola Curry[39], Endre Czeiter[24], Marek Czosnyka[26], Claire Dahyot-Fizelier[40], Helen Dawes[41], Véronique De Keyser[42], Vincent Degos[16], Francesco Della Corte[43], Hugo den Boogert[10], Bart Depreitere[44], Đula Đilvesi [45], Abhishek Dixit[46], Emma Donoghue[22], Jens Dreier[47], Guy-Loup Dulière[48], Ari Ercole[46], Patrick Esser[41], Erzsébet Ezer[49], Martin Fabricius[50], Valery L. Feigin[51], Kelly Foks[52], Shirin Frisvold[53], Alex Furmanov[54], Pablo Gagliardo[55], Damien Galanaud[16], Dashiell Gantner[28], Guoyi Gao[56], Pradeep George[57], Alexandre Ghuysen[58], Lelde Giga[59], Ben Glocker[60], Jagoš Golubovic[45], Pedro A. Gomez [61], Johannes Gratz[62], Benjamin Gravesteijn[33], Francesca Grossi[43], Russell L. Gruen[63], Deepak Gupta[64], Juanita A. Haagsma[33], Iain Haitsma[65], Raimund Helbok[13], Eirik Helseth[66], Lindsay Horton [67], Jilske Huijben[33], Peter J. Hutchinson[68], Bram Jacobs[69], Stefan Jankowski[70], Mike Jarrett[21], Ji-yao Jiang[56], Kelly Jones[51], Mladen Karan[47], Angelos G. Kolias[68], Erwin Kompanje[71], Daniel Kondziella[50], Evgenios Koraropoulos[46], Lars-Owe Koskinen[72], Noémi Kovács[73], Alfonso Lagares[61], Linda Lanyon[57], Steven Laureys[74], Fiona Lecky[75], Rolf Lefering[76], Valerie Legrand[77], Aurelie Lejeune[78], Leon Levi[79], Roger Lightfoot[80], Hester Lingsma[33], Andrew I.R. Maas[42], Ana M. Castaño-León[61], Marc Maegele[81], Marek Majdan[20], Alex Manara[82], Geoffrey Manley[83], Costanza Martino[84], Hugues Maréchal[48], Julia Mattern[85], Catherine McMahon[86], Béla Melegh[87], David Menon[46], Tomas Menovsky[42], Davide Mulazzi[27], Visakh Muraleedharan[57], Lynnette Murray[28], Nandesh Nair[42], Ancuta Negru[88], David Nelson[1], Virginia Newcombe[46], Daan Nieboer[33], Quentin Noirhomme[74], József Nyirádi[2], Otesile Olubukola[75], Matej Oresic[89], Fabrizio Ortolano[27], Aarno Palotie[90, 91, 92], Paul M. Parizel[93], Jean-François Payen[94], Natascha Perera[12], Vincent Perlbarg[16], Paolo Persona[95], Wilco Peul[96], Anna Piippo-Karjalainen[97], Matti Pirinen[90], Horia Ples[88], Suzanne Polinder[33], Inigo Pomposo[29], Jussi P. Posti [98], Louis Puybasset[99], Andreea Radoi [100], Arminas Ragauskas[101], Rahul Raj[97], Malinka Rambadagalla[102], Ruben Real[38], Jonathan Rhodes[103], Sylvia Richardson[104], Sophie Richter[46], Samuli Ripatti[90], Saulius Rocka[101], Cecilie Roe[105], Olav Roise[106], Jonathan Rosand[107], Jeffrey V. Rosenfeld[108], Christina Rosenlund[109], Guy Rosenthal[54], Proffesor Clusmann[34], Sandra Rossi[95], Daniel Rueckert[60], Martin Rusnák[110], Juan Sahuquillo[100], Oliver Sakowitz[85, 111], Renan Sanchez-Porras[111], Janos Sandor[112], Nadine Schäfer[76], Silke Schmidt[113], Herbert Schoechl[114], Guus Schoonman[115], Rico Frederik Schou[116], Elisabeth Schwendenwein[6], Charlie Sewalt[33], Toril Skandsen[117, 118] , Peter Smielewski[26], Abayomi Sorinola[119], Emmanuel Stamatakis[46], Simon Stanworth[39], Ana Stevanovic[34], Robert Stevens[120], William Stewart[121], Ewout W. Steyerberg[33, 122], Nino Stocchetti[123], Nina Sundström[124], Anneliese Synnot[22, 125], Riikka Takala[126], Viktória Tamás[119], Tomas Tamosuitis[127], Mark Steven Taylor[20], Braden Te Ao[51], Olli Tenovuo[98], Alice Theadom[51], Matt Thomas[82], Dick Tibboel[128], Marjolein Timmers[71], Christos Tolias[129], Tony Trapani[28], Cristina Maria Tudora[88], Peter Vajkoczy [130], Shirley Vallance[28], Egils Valeinis[59], Zoltán Vámos[49], Gregory Van der Steen[42], Joukje van der Naalt[69], Jeroen T.J.M. van Dijck [96], Thomas A. van Essen[96], Wim Van Hecke[131], Caroline van Heugten[132], Dominique Van Praag[133], Thijs Vande Vyvere[131], Audrey Vanhaudenhuyse[16, 74], Roel P. J. van Wijk[97], Alessia Vargiolu[32], Emmanuel Vega[79], Kimberley Velt[33], Jan Verheyden[131], Paul M. Vespa[134], Anne Vik[117, 135], Rimantas Vilcinis[127], Victor Volovici[65], Nicole von Steinbüchel[38], Daphne Voormolen[33], Petar Vulekovic[45], Kevin K.W. Wang[136], Eveline Wiegers[33], Guy Williams[46], Lindsay Wilson[67], Stefan Winzeck[46], Stefan Wolf[137], Zhihui Yang[136], Peter Ylén[138], Alexander Younsi[85], Frederik A. Zeiler[46,139], Veronika Zelinkova[20], Agate Ziverte[59] , Tommaso Zoerle[27]

[1] Department of Physiology and Pharmacology, Section of Perioperative Medicine and Intensive Care, Karolinska Institutet, Stockholm, Sweden
[2] János Szentágothai Research Centre, University of Pécs, Pécs, Hungary
[3] Division of Surgery and Clinical Neuroscience, Department of Physical Medicine and Rehabilitation, Oslo University Hospital and University of Oslo, Oslo, Norway
[4] Department of Neurosurgery, University Hospital Northern Norway, Tromso, Norway
[5] Department of Physical Medicine and Rehabilitation, University Hospital Northern Norway, Tromso, Norway
[6] Trauma Surgery, Medical University Vienna, Vienna, Austria
[7] Department of Anesthesiology & Intensive Care, University Hospital Nancy, Nancy, France
[8] Raymond Poincare hospital, Assistance Publique – Hopitaux de Paris, Paris, France
[9] Department of Anesthesiology & Intensive Care, S Raffaele University Hospital, Milan, Italy
[10] Department of Neurosurgery, Radboud University Medical Center, Nijmegen, The Netherlands
[11] Department of Neurosurgery, University of Szeged, Szeged, Hungary
[12] International Projects Management, ARTTIC, Munchen, Germany
[13] Department of Neurology, Neurological Intensive Care Unit, Medical University of Innsbruck, Innsbruck, Austria
[14] Department of Neurosurgery & Anesthesia & intensive care medicine, Karolinska University Hospital, Stockholm, Sweden
[15] NIHR Surgical Reconstruction and Microbiology Research Centre, Birmingham, UK
[16] Anesthesie-Réanimation, Assistance Publique – Hopitaux de Paris, Paris, France
[17] Department of Anesthesia & ICU, AOU Città della Salute e della Scienza di Torino - Orthopedic and Trauma Center, Torino, Italy
[18] Department of Neurology, Odense University Hospital, Odense, Denmark
[19] BehaviourWorks Australia, Monash Sustainability Institute, Monash University, Victoria, Australia
[20] Department of Public Health, Faculty of Health Sciences and Social Work, Trnava University, Trnava, Slovakia
[21] Quesgen Systems Inc., Burlingame, California, USA
[22] Australian & New Zealand Intensive Care Research Centre, Department of Epidemiology and Preventive Medicine, School of Public Health and Preventive Medicine, Monash University, Melbourne, Australia
[23] Department of Surgery and Perioperative Science, Umeå University, Umeå, Sweden
[24] Department of Neurosurgery, Medical School, University of Pécs, Hungary and Neurotrauma Research Group, János Szentágothai Research Centre, University of Pécs, Hungary
[25] Department of Medical Psychology, Universitätsklinikum Hamburg-Eppendorf, Hamburg, Germany
[26] Brain Physics Lab, Division of Neurosurgery, Dept of Clinical Neurosciences, University of Cambridge, Addenbrooke's Hospital, Cambridge, UK
[27] Neuro ICU, Fondazione IRCCS Cà Granda Ospedale Maggiore Policlinico, Milan, Italy
[28] ANZIC Research Centre, Monash University, Department of Epidemiology and Preventive Medicine, Melbourne, Victoria, Australia
[29] Department of Neurosurgery, Hospital of Cruces, Bilbao, Spain
[30] NeuroIntensive Care, Niguarda Hospital, Milan, Italy
[31] School of Medicine and Surgery, Università Milano Bicocca, Milano, Italy
[32] NeuroIntensive Care, ASST di Monza, Monza, Italy
[33] Department of Public Health, Erasmus Medical Center-University Medical Center, Rotterdam, The Netherlands
[34] Department of Neurosurgery, University Hospital of Aachen, Aachen, Germany
[35] Department of Anesthesia & Neurointensive Care, Cambridge University Hospital NHS Foundation Trust, Cambridge, UK
[36] School of Public Health & PM, Monash University and The Alfred Hospital, Melbourne, Victoria, Australia

[37] Radiology/MRI department, MRC Cognition and Brain Sciences Unit, Cambridge, UK
[38] Institute of Medical Psychology and Medical Sociology, Universitätsmedizin Göttingen, Göttingen, Germany
[39] Oxford University Hospitals NHS Trust, Oxford, UK
[40] Intensive Care Unit, CHU Poitiers, Potiers, France
[41] Movement Science Group, Faculty of Health and Life Sciences, Oxford Brookes University, Oxford, UK
[42] Department of Neurosurgery, Antwerp University Hospital and University of Antwerp, Edegem, Belgium
[43] Department of Anesthesia & Intensive Care, Maggiore Della Carità Hospital, Novara, Italy
[44] Department of Neurosurgery, University Hospitals Leuven, Leuven, Belgium
[45] Department of Neurosurgery, Clinical centre of Vojvodina, Faculty of Medicine, University of Novi Sad, Novi Sad, Serbia
[46] Division of Anaesthesia, University of Cambridge, Addenbrooke's Hospital, Cambridge, UK
[47] Center for Stroke Research Berlin, Charité – Universitätsmedizin Berlin, corporate member of Freie Universität Berlin, Humboldt-Universität zu Berlin, and Berlin Institute of Health, Berlin, Germany
[48] Intensive Care Unit, CHR Citadelle, Liège, Belgium
[49] Department of Anaesthesiology and Intensive Therapy, University of Pécs, Pécs, Hungary
[50] Departments of Neurology, Clinical Neurophysiology and Neuroanesthesiology, Region Hovedstaden Rigshospitalet, Copenhagen, Denmark
[51] National Institute for Stroke and Applied Neurosciences, Faculty of Health and Environmental Studies, Auckland University of Technology, Auckland, New Zealand
[52] Department of Neurology, Erasmus MC, Rotterdam, the Netherlands
[53] Department of Anesthesiology and Intensive care, University Hospital Northern Norway, Tromso, Norway
[54] Department of Neurosurgery, Hadassah-hebrew University Medical center, Jerusalem, Israel
[55] Fundación Instituto Valenciano de Neurorrehabilitación (FIVAN), Valencia, Spain
[56] Department of Neurosurgery, Shanghai Renji hospital, Shanghai Jiaotong University/school of medicine, Shanghai, China
[57] Karolinska Institutet, INCF International Neuroinformatics Coordinating Facility, Stockholm, Sweden
[58] Emergency Department, CHU, Liège, Belgium
[59] Neurosurgery clinic, Pauls Stradins Clinical University Hospital, Riga, Latvia
[60] Department of Computing, Imperial College London, London, UK
[61] Department of Neurosurgery, Hospital Universitario 12 de Octubre, Madrid, Spain
[62] Department of Anesthesia, Critical Care and Pain Medicine, Medical University of Vienna, Austria
[63] College of Health and Medicine, Australian National University, Canberra, Australia
[64] Department of Neurosurgery, Neurosciences Centre & JPN Apex trauma centre, All India Institute of Medical Sciences, New Delhi-110029, India
[65] Department of Neurosurgery, Erasmus MC, Rotterdam, the Netherlands
[66] Department of Neurosurgery, Oslo University Hospital, Oslo, Norway
[67] Division of Psychology, University of Stirling, Stirling, UK
[68] Division of Neurosurgery, Department of Clinical Neurosciences, Addenbrooke's Hospital & University of Cambridge, Cambridge, UK
[69] Department of Neurology, University of Groningen, University Medical Center Groningen, Groningen, Netherlands
[70] Neurointensive Care , Sheffield Teaching Hospitals NHS Foundation Trust, Sheffield, UK
[71] Department of Intensive Care and Department of Ethics and Philosophy of Medicine, Erasmus Medical Center, Rotterdam, The Netherlands
[72] Department of Clinical Neuroscience, Neurosurgery, Umeå University, Umeå, Sweden
[73] Hungarian Brain Research Program - Grant No. KTIA_13_NAP-A-II/8, University of Pécs, Pécs, Hungary

[74] Cyclotron Research Center , University of Liège, Liège, Belgium
[75] Emergency Medicine Research in Sheffield, Health Services Research Section, School of Health and Related Research (ScHARR), University of Sheffield, Sheffield, UK
[76] Institute of Research in Operative Medicine (IFOM), Witten/Herdecke University, Cologne, Germany
[77] VP Global Project Management CNS, ICON, Paris, France
[78] Department of Anesthesiology-Intensive Care, Lille University Hospital, Lille, France
[79] Department of Neurosurgery, Rambam Medical Center, Haifa, Israel
[80] Department of Anesthesiology & Intensive Care, University Hospitals Southhampton NHS Trust, Southhampton, UK
[81] Cologne-Merheim Medical Center (CMMC), Department of Traumatology, Orthopedic Surgery and Sportmedicine, Witten/Herdecke University, Cologne, Germany
[82] Intensive Care Unit, Southmead Hospital, Bristol, Bristol, UK
[83] Department of Neurological Surgery, University of California, San Francisco, California, USA
[84] Department of Anesthesia & Intensive Care,M. Bufalini Hospital, Cesena, Italy
[85] Department of Neurosurgery, University Hospital Heidelberg, Heidelberg, Germany
[86] Department of Neurosurgery, The Walton centre NHS Foundation Trust, Liverpool, UK
[87] Department of Medical Genetics, University of Pécs, Pécs, Hungary
[88] Department of Neurosurgery, Emergency County Hospital Timisoara , Timisoara, Romania
[89] School of Medical Sciences, Örebro University, Örebro, Sweden
[90] Institute for Molecular Medicine Finland, University of Helsinki, Helsinki, Finland
[91] Analytic and Translational Genetics Unit, Department of Medicine; Psychiatric & Neurodevelopmental Genetics Unit, Department of Psychiatry; Department of Neurology, Massachusetts General Hospital, Boston, MA, USA
[92] Program in Medical and Population Genetics; The Stanley Center for Psychiatric Research, The Broad Institute of MIT and Harvard, Cambridge, MA, USA
[93] Department of Radiology, Antwerp University Hospital and University of Antwerp, Edegem, Belgium
[94] Department of Anesthesiology & Intensive Care, University Hospital of Grenoble, Grenoble, France
[95] Department of Anesthesia & Intensive Care, Azienda Ospedaliera Università di Padova, Padova, Italy
[96] Dept. of Neurosurgery, Leiden University Medical Center, Leiden, The Netherlands and Dept. of Neurosurgery, Medical Center Haaglanden, The Hague, The Netherlands
[97] Department of Neurosurgery, Helsinki University Central Hospital
[98] Division of Clinical Neurosciences, Department of Neurosurgery and Turku Brain Injury Centre, Turku University Hospital and University of Turku, Turku, Finland
[99] Department of Anesthesiology and Critical Care, Pitié -Salpêtrière Teaching Hospital, Assistance Publique, Hôpitaux de Paris and University Pierre et Marie Curie, Paris, France
[100] Neurotraumatology and Neurosurgery Research Unit (UNINN), Vall d'Hebron Research Institute, Barcelona, Spain
[101] Department of Neurosurgery, Kaunas University of technology and Vilnius University, Vilnius, Lithuania
[102] Department of Neurosurgery, Rezekne Hospital, Latvia
[103] Department of Anaesthesia, Critical Care & Pain Medicine NHS Lothian & University of Edinburg, Edinburgh, UK
[104] Director, MRC Biostatistics Unit, Cambridge Institute of Public Health, Cambridge, UK
[105] Department of Physical Medicine and Rehabilitation, Oslo University Hospital/University of Oslo, Oslo, Norway
[106] Division of Surgery and Clinical Neuroscience, Oslo University Hospital, Oslo, Norway
[107] Broad Institute, Cambridge MA Harvard Medical School, Boston MA, Massachusetts General Hospital, Boston MA, USA

[108] National Trauma Research Institute, The Alfred Hospital, Monash University, Melbourne, Victoria, Australia
[109] Department of Neurosurgery, Odense University Hospital, Odense, Denmark
[110] International Neurotrauma Research Organisation, Vienna, Austria
[111] Klinik für Neurochirurgie, Klinikum Ludwigsburg, Ludwigsburg, Germany
[112] Division of Biostatistics and Epidemiology, Department of Preventive Medicine, University of Debrecen, Debrecen, Hungary
[113] Department Health and Prevention, University Greifswald, Greifswald, Germany
[114] Department of Anaesthesiology and Intensive Care, AUVA Trauma Hospital, Salzburg, Austria
[115] Department of Neurology, Elisabeth-TweeSteden Ziekenhuis, Tilburg, the Netherlands
[116] Department of Neuroanesthesia and Neurointensive Care, Odense University Hospital, Odense, Denmark
[117] Department of Neuromedicine and Movement Science, Norwegian University of Science and Technology, NTNU, Trondheim, Norway
[118] Department of Physical Medicine and Rehabilitation, St.Olavs Hospital, Trondheim University Hospital, Trondheim, Norway
[119] Department of Neurosurgery, University of Pécs, Pécs, Hungary
[120] Division of Neuroscience Critical Care, John Hopkins University School of Medicine, Baltimore, USA
[121] Department of Neuropathology, Queen Elizabeth University Hospital and University of Glasgow, Glasgow, UK
[122] Dept. of Department of Biomedical Data Sciences, Leiden University Medical Center, Leiden, The Netherlands
[123] Department of Pathophysiology and Transplantation, Milan University, and Neuroscience ICU, Fondazione IRCCS Cà Granda Ospedale Maggiore Policlinico, Milano, Italy
[124] Department of Radiation Sciences, Biomedical Engineering, Umeå University, Umeå, Sweden
[125] Cochrane Consumers and Communication Review Group, Centre for Health Communication and Participation, School of Psychology and Public Health, La Trobe University, Melbourne, Australia
[126] Perioperative Services, Intensive Care Medicine and Pain Management, Turku University Hospital and University of Turku, Turku, Finland
[127] Department of Neurosurgery, Kaunas University of Health Sciences, Kaunas, Lithuania
[128] Intensive Care and Department of Pediatric Surgery, Erasmus Medical Center, Sophia Children's Hospital, Rotterdam, The Netherlands
[129] Department of Neurosurgery, Kings college London, London, UK
[130] Neurologie, Neurochirurgie und Psychiatrie, Charité – Universitätsmedizin Berlin, Berlin, Germany
[131] icoMetrix NV, Leuven, Belgium
[132] Movement Science Group, Faculty of Health and Life Sciences, Oxford Brookes University, Oxford, UK
[133] Psychology Department, Antwerp University Hospital, Edegem, Belgium
[134] Director of Neurocritical Care, University of California, Los Angeles, USA
[135] Department of Neurosurgery, St.Olavs Hospital, Trondheim University Hospital, Trondheim, Norway
[136] Department of Emergency Medicine, University of Florida, Gainesville, Florida, USA
[137] Department of Neurosurgery, Charité – Universitätsmedizin Berlin, corporate member of Freie Universität Berlin, Humboldt-Universität zu Berlin, and Berlin Institute of Health, Berlin, Germany
[138] VTT Technical Research Centre, Tampere, Finland
[139] Section of Neurosurgery, Department of Surgery, Rady Faculty of Health Sciences, University of Manitoba, Winnipeg, MB, Canada

Table S1. Method of administration of the Rivermead Post-Concussion Symptoms Questionnaire.

	Telephone Interview	Postal Questionnaire	Web-Based Completion	Face-to-Face Interview	Not Completed/Missing
3 months	97 (3.3%)	784 (26.5%)	2 (0.07%)	418 (14.1%)	1653 (55.9%)
6 months	45 (1.5%)	594 (20.1%)	4 (0.14%)	659 (22.3%)	1653 (55.9%)

Table S2. Characteristics of mTBI patients with completed and uncompleted RPQ.

	Complete RPQ	Incomplete RPQ	*P*-Value
N	1302	1653	
Gender (male)	827 (63.5%)	1076 (65.1%)	0.374
Age [1] (years)	53 (35–66)	49 (29–69)	0.207
Education [1] (years)	14 (11–17)	12 (10–15)	<0.01
Injury Mechanism			<0.01
Road traffic accident	504 (38.7%)	506 (30.6%)	
Incidental fall	616 (47.3%)	825 (49.9%)	
Other non-intentional	72 (5.5%)	91 (5.5%)	
Violence/assault	43 (3.3%)	140 (8.5%)	
Act of mass violence	1 (0.1%)	3 (0.2%)	
Suicide attempt	7 (0.5%)	8 (0.5%)	
Other	42 (3.2%)	50 (3.0%)	
Unknown	17 (1.3%)	30 (1.8%)	
Psychiatric Medical History	146 (11.2%)	249 (15.1%)	<0.01
Anxiety	36 (24.7%)	77 (30.9%)	0.183
Depression	89 (61.0%)	146 (58.6%)	0.650
Sleep disorders	19 (13.0%)	34 (13.7%)	0.857
Schizophrenia	3 (2.1%)	2 (0.8%)	0.283
Substance abuse disorder	17 (11.6%)	62 (24.9%)	<0.01
Other	19 (13.0%)	43 (17.3%)	0.262
GCS baseline [1]	15 (15–15)	15 (14–15)	0.037
Computed Tomography			
Any intracranial injury on first CT	599 (46.0%)	652 (39.4%)	<0.01

[1] Data are displayed as median, with the first and third quartile given within brackets. mTBI, mild traumatic brain injury; RPQ, Rivermead Post-Concussion Symptoms Questionnaire; CT, Computed Tomography.

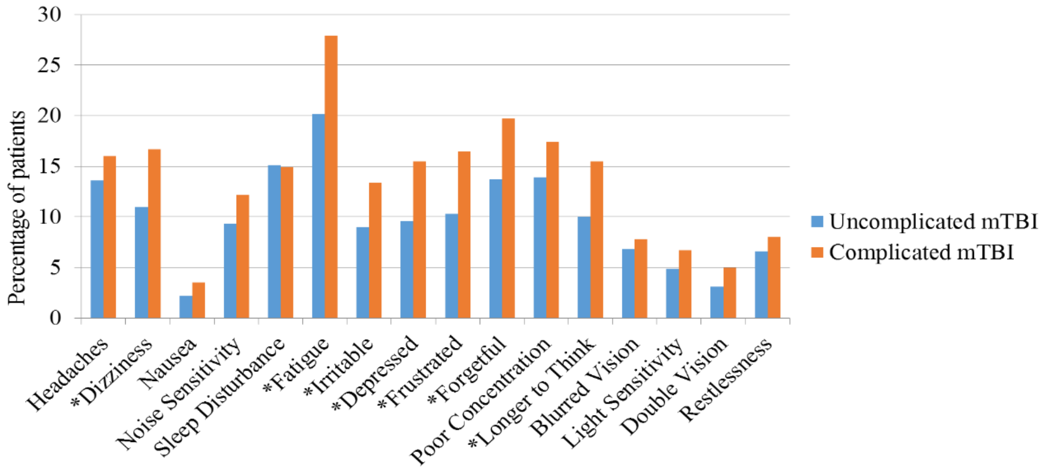

(a)

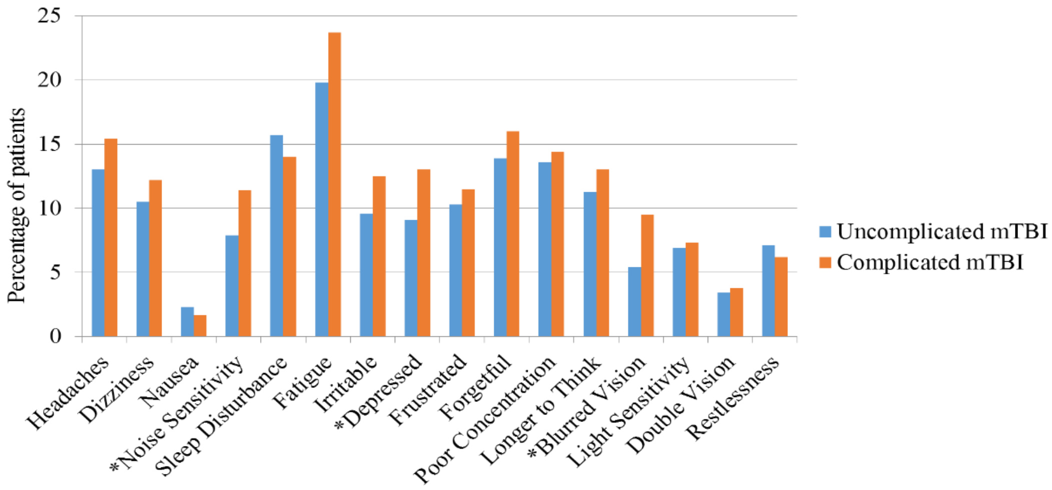

(b)

Figure S1. (**a**) Frequency of post-concussion symptoms with a severity rating of 3 (moderate problem) or higher at 3 months. (**b**) Frequency of post-concussion symptoms with a severity rating of 3 (moderate problem) or higher at 6 months. * significant ($p < 0.05$).

Table S3. Significant predictors in a multivariable model on the association of complicated versus uncomplicated mTBI on the presence of PCS (with a severity rating of 2 (mild problem) or higher).

**Post-Concussion Syndrome**	**3 Months**			**6 Months**		
**Predictor**	**OR**	**95% CI**	***P*-Value**	**OR**	**95% CI**	***P*-Value**
Age	0.995	0.989–1.001	0.083	1.002	0.996–1.009	0.446
Gender (male)	0.588	0.460–0.749	<0.01	0.617	0.483–0.790	<0.01
Education	0.998	0.970–1.027	0.894	0.979	0.950–1.010	0.184
Stratum (ER)						
Admission	1.517	1.127–2.044	<0.01	1.254	0.927–1.670	0.142
ICU	2.013	1.371–2.956	<0.01	2.128	1.440–3.143	<0.01
GCS baseline	0.925	0.753–1.137	0.459	0.927	0.753–1.142	0.477
Injury Mechanism (other)						
Road traffic accident	1.105	0.722–1.692	0.644	1.045	0.672–1.623	0.845
Incidental fall	1.252	0.967–1.620	0.088	1.623	1.249–2.110	<0.01
Violence/assault/suicide	1.012	0.543–1.888	0.968	1.353	0.731–2.505	0.336
Psychiatric Medical History (yes)	1.592	0.698–3.632	0.269	2.800	1.232–6.365	0.014
Anxiety (yes)	1.198	0.516–2.783	0.674	0.687	0.299–1.577	0.376
Depression (yes)	1.146	0.513–2.559	0.740	0.832	0.374–1.853	0.653
Sleep disorders (yes)	1.631	0.557–4.782	0.372	0.817	0.283–2.357	0.709
Substance abuse disorder (yes)	1.272	0.414–3.915	0.674	2.092	0.628–6.968	0.229
Other	1.063	0.354–3.188	0.913	0.509	0.168–1.539	0.232
Any intracranial injury on first CT (yes)	1.252	0.945–1.658	0.117	1.084	0.807–1.456	0.594
N	1302			1302		
Nagelkerke R2	0.072			0.085		

mTBI, mild traumatic brain injury; PCS, post-concussion syndrome; OR, Odds Ratio; 95% CI, 95% confidence interval; PCS, post-concussion syndrome; ER, emergency room; ICU, intensive care unit; RTA, road traffic accident; CT, computed tomography. Note: schizophrenia was taken out of the analyses due to limited number of patients; violence/assault, act of mass violence and suicide attempt were grouped together into "violence/assault/suicide"; other non-intentional and other were grouped together into "other".
